# Neural Code—*Neural Self-information Theory* on How Cell-Assembly Code Rises from Spike Time and Neuronal Variability

**DOI:** 10.3389/fncel.2017.00236

**Published:** 2017-08-30

**Authors:** Meng Li, Joe Z. Tsien

**Affiliations:** ^1^Brain and Behavior Discovery Institute, Medical College of Georgia, Augusta University Augusta, GA, United States; ^2^The Brain Decoding Center, BanNa Biomedical Research Institute, Yunnan Academy of Science and Technology Yunnan Province, China

**Keywords:** neural code, self-information, neural computing, neural spike variability, variability-surprisal, surprisal code, cell assembly, code of silence

## Abstract

A major stumbling block to cracking the real-time neural code is neuronal variability - neurons discharge spikes with enormous variability not only across trials within the same experiments but also in resting states. Such variability is widely regarded as a noise which is often deliberately averaged out during data analyses. In contrast to such a dogma, we put forth the *Neural Self-Information Theory* that neural coding is operated based on the self-information principle under which variability in the time durations of inter-spike-intervals (ISI), or neuronal silence durations, is self-tagged with discrete information. As the self-information processor, each ISI carries a certain amount of information based on its variability-probability distribution; higher-probability ISIs which reflect the balanced excitation-inhibition *ground state* convey minimal information, whereas lower-probability ISIs which signify rare-occurrence surprisals in the form of extremely transient or prolonged silence carry most information. These variable silence durations are naturally coupled with intracellular biochemical cascades, energy equilibrium and dynamic regulation of protein and gene expression levels. As such, this *silence variability-based self-information code* is completely intrinsic to the neurons themselves, with no need for outside observers to set any reference point as typically used in the *rate code, population code* and *temporal code* models. Moreover, temporally coordinated ISI surprisals across cell population can inherently give rise to robust real-time cell-assembly codes which can be readily sensed by the downstream neural clique assemblies. One immediate utility of this *self-information code* is a general decoding strategy to uncover a variety of cell-assembly patterns underlying external and internal categorical or continuous variables in an unbiased manner.

Two hard problems lie at the heart of brain decoding research; namely, what is ***the basic wiring logic of the brain?*** And *what is* ***the basic operational rule for representing real-time information?*** With 86 billion neurons and 100 trillion synaptic connections in the human brain, it is conceivable that the understanding of the brain's basic wiring logic is the foundation upon which dynamic coding of cognitive information can be meaningfully executed (Hebb, 1949; Brenner and Sejnowski, 2011; Tsien, 2015a,b). In the absence of such overarching framework under which neurons connect or organize themselves, merely reading out neural signals corresponding to external stimulus identity is very much like a fictional biologist who may discern a foreign message from a radio yet has no idea about how radios work. We refer to this *Connectivity Logic* problem as “***The Dead Brain's problem***,” because the wiring logic in a live brain would remain the same even if the brain suddenly died (or were dropped into a liquid nitrogen tank). Emerging studies have revealed that neural clique assemblies—the brain's basic computational motif—are organized via the power-of-two-based permutation logic to generate not only specific perceptions and memories, but also generalized knowledge and adaptive behaviors (Tsien, 2015a,b; Xie et al., 2016). Along this line of investigation, there are many fascinating questions as to how development and evolution might use various components to construct complex neural circuits and the brains across a wide range of animal species to give rise to both specific and general functions (Rakic, 2009; Grillner et al., 2013; Tsien et al., 2013; Defelipe, 2015; Kiehn, 2016; Tsien, 2016).

The second hard problem relates to *cracking neural code*—the rule under which information is signaled inside the brain in real time. We refer it as “***The Live Brain's problem***,” that is, how information is dynamically represented by patterns of action potential, or spike, generated by neurons in relevant brain regions corresponding to moment-to-moment perceptions, memories, creative thoughts and behaviors (Tulving, 1972; Squire and Zola, 1998; Brown et al., 2004; Tsien, 2007; Zhang et al., 2013; Kiehn, 2016). This effort in examining how spike patterns signal stimulus identity has often been dubbed as “*to crack neural code*.”

The search for neural code has its long history due to Edgar Adrian and Yngve Zotterman's original observation in the 1920s that sensory nerves innervating muscle emitted more spikes in response to increased amounts of weight hung from a muscle (Adrian and Zotterman, 1926). This landmark work has established the central dogma that neurons encode information by changing firing rates. Yet, the stumbling block in cracking the real-time neural code is neuronal variability. Over the decades, researchers have realized that neurons in every type of neural circuit—whether they are engaged in processing information such as touch, smell, vision, hearing, motor action or spatial navigation, or associative memories, etc.—discharge spikes all the time with tremendous variability in both the “control” resting states (including awake or sleep) and across trials within the same experiments (Figure 1A; Werner and Mountcastle, 1963; Poggio and Viernstein, 1964; Ratliff et al., 1968; Georgopoulos et al., 1986; Shadlen and Newsome, 1994; Fenton and Muller, 1998; Stein et al., 2005; Churchland et al., 2010). This notorious spike variability has made the *rate code* and *temporal code* models ill-suited to reliably predict stimulus identity on a moment-to-moment basis (Eggermont, 1998; Fenton and Muller, 1998; Faisal et al., 2008). The current dogma is that firing variability reflects noise or is a nuisance to outside observers. This view is reflected in popular practice by averaging spike trains over multiple trials, such as peri-stimulus time histogram (PSTH). Although, such an averaging approach is useful to characterize the tuning properties of the recorded neurons, it is generally agreed that it bears no resemblance to how neurons would signal information in real time.

**Figure 1:**
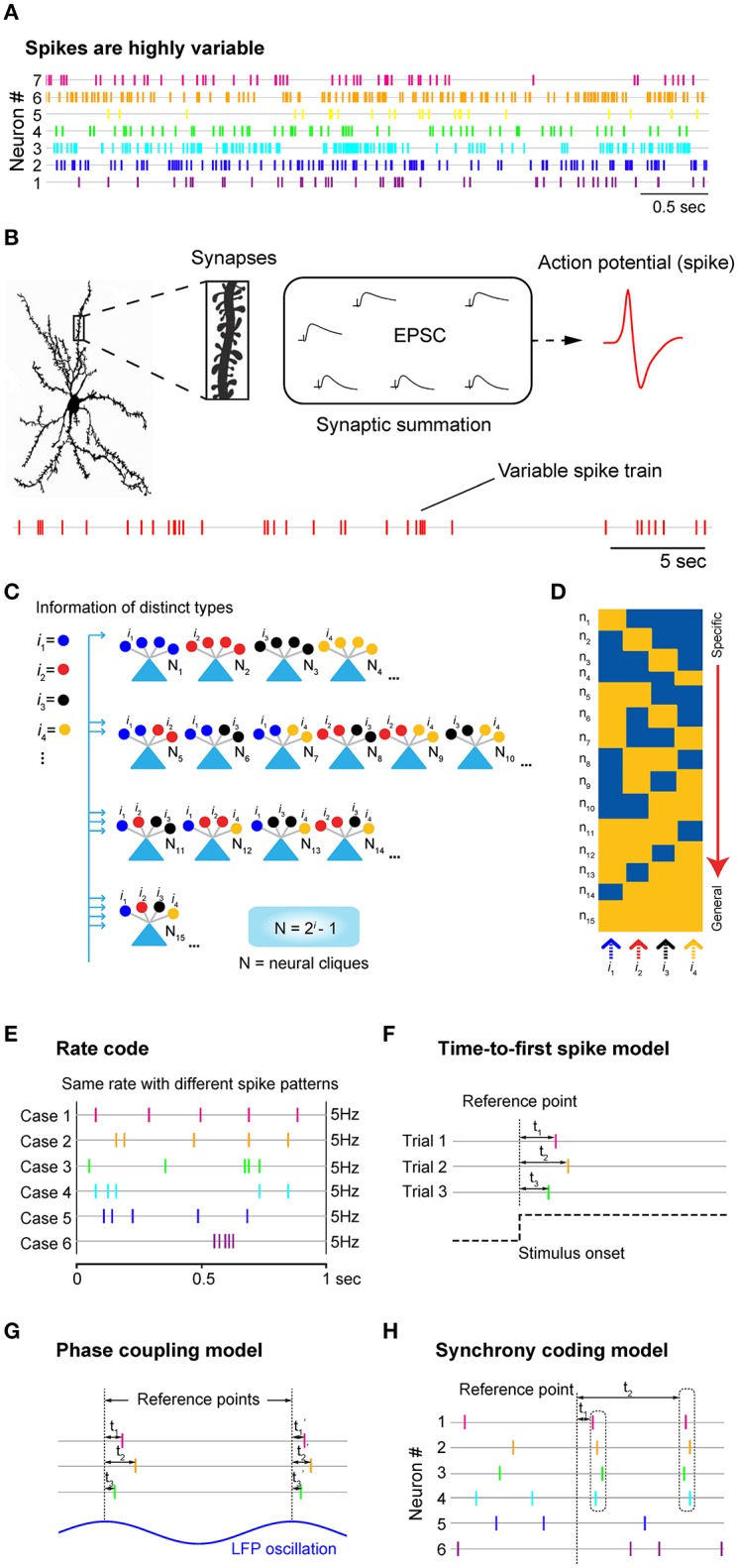
Neuronal variability, underlying logic at synaptic and cell-assembly levels, and the traditional neural coding models. **(A)** Neurons discharge spikes all the time with enormous variability. Spike trains shown here are simultaneously recorded seven units from mice prefrontal cortex during animal's quiet-awake period using tetrodes. **(B)** A cortical neuron may contain tens of thousands of synapses which can contribute to changes in excitatory postsynaptic potential (EPSP), leading to the generation of action potential or spike at the soma. Stochastic nature of synaptic patterns leads to highly variable spike trains in both the resting “control” condition and stimulus-presentation experiments. **(C)** Power-of-two-based Cell-Assembly Wiring Logic as the brain's basic functional computational motif (FCM). A schematic illustration of a power-of-two connectivity motif consisted of 15 distinct neural cliques (N1-15) based on all the possible connectivity patterns for processing 4 distinct inputs (*i* = 4). **(D)** This motif gives rise to a specific-to-general feature extraction assembly. **(E)** The rate code model emphasizes the number of spikes within a defined time window, while ignoring the temporal structures in spike patterns. Five examples of the same firing rate (5 Hz) with completely different spike patterns were used for illustration. **(F)** The time-to-first-spike model of the temporal code emphasizes that key information is encoded in the relative arrival time of the first spike after stimulus onset. **(G)** The phase-coupling model focused on the temporal relationship between spike changes and local field potential (LFP) oscillation phases. **(H)** The synchrony code proposed that information coding and binding were achieved by *some “uniquely meaningful” spikes* which were transiently synchronized among different cells. In all cases, the rate code, population code, and temporal code models require a reference point (i.e., time zeros of stimulation, or oscillation phase, etc.) for data analyses. As such, these approaches are generally known as the biased methods. Panels **(E–H)** are artistic illustrations for better visualizing the four popular coding models.

## Does neuronal variability reflect noise or something else?

Currently, two schools of thoughts come to describe what neuronal variability stands for. The first one is the widely held view that firing fluctuations in neurons reflect noise derived from molecular, synaptic, and circuitry levels (Eggermont, 1998; Ermentrout et al., 2008; Faisal et al., 2008; Masquelier, 2013). This view has led to intense studies of the source and degree of noise in experiments and simulations (Shadlen and Newsome, 1994; Stein et al., 2005; Faisal et al., 2008; Rolls and Deco, 2010; Boerlin and Deneve, 2011; Hartmann et al., 2015).

The second view is that neuronal variability is not entirely noise, rather it may also contain uncontrolled internal variables influenced by attention or intent, because the observed noise seems to be correlated within the recorded population (measured as noise correlation; Lee et al., 1998; Churchland et al., 2010; Marcos et al., 2013; Lin et al., 2015). A series of studies also suggested that neuronal variability can be beneficial for boosting weak signals (Stacey and Durand, 2001) or serving as modulatory signals (Lee et al., 1998; Boerlin and Deneve, 2011; Kohn et al., 2016; Saberi-Moghadam et al., 2016).

While the question of whether neuronal variability reflects noise or unidentified modulatory signals is still under debate, it is known that neurons are capable of generating precisely-timed spikes in response to fluctuating currents injected at the soma (Mainen and Sejnowski, 1995; Abbott and Sejnowski, 1999; Toups et al., 2012). Thus, spike variability is not due to imprecision in spike generation at the soma *per se*. At the structural and conceptual levels, it is not very difficult to appreciate why such variability should be fully expected. Neurons in the mammalian brain contain many thousands of synaptic connections, ranging from ~30,000 synapses per pyramidal cell in the neocortex up to 200,000 synapses per Purkinje cell in the cerebellum (Andersen, 1990; Megias et al., 2001; Guillery, 2005; Herculano-Houzel, 2009; Defelipe, 2015). Summation of these postsynaptic currents triggers action potentials in the postsynaptic cell soma (Figure 1B). With ongoing synaptic inputs coming from tens of thousands of excitatory synapses, as well as hundreds or thousands of highly localized inhibitory synapses (Klausberger and Somogyi, 2008), spike emission in any given neuron would be stochastic with enormous variability. In addition, other mechanisms and factors (i.e., slow neurotransmitters, hormonal peptides, and activity-dependent modulation of non-receptor ion channels) can also exert great influence on synaptic and membrane properties (Hille, 2001; Brenner et al., 2005; Gu et al., 2007) leading to the variability in spike train.

## Why does a neuron need tens of thousands of synapses? binding vs. approximation?

Theoretically, an increased number of synapses would permit neurons to possess greater *information binding capacity*. This seems to make perfect sense when synapse numbers were at a smaller scale, say, with several dozen synapses each processing distinct features so that binding can be performed (to form complex “grandmother cells” encoding a face, famous people or a nest; Gross et al., 2002; Quiroga et al., 2005; Lin et al., 2007). A recent analysis of face patches in the monkey brain suggests that face cells may only utilize 50 or less dimensional facial features (Chang and Tsao, 2017). Therefore, we would argue that with 30,000 or more synapses per pyramidal neuron, the underlying logic may go beyond the mere feature integration and information-binding.

What good are tens of thousands of synapses? We would like to suggest that the major purpose of such an elaborate structural arrangement is to achieve *maximal feature approximation and efficient utilization* of the evolutionarily selected neural clique assemblies. This maximal approximation strategy is necessary for the brain to best utilize the developmentally pre-configured *functional computational motifs* (FCM) described by the *Theory of Connectivity* (Tsien, 2015a,b). The theory posits that as the basic computational unit of the brain, FCM organizes its principal cell assemblies via the power-of-two-based permutation logic to form a comprehensive set of specific-to-general neural-clique assemblies (*N* = 2^*i*^-1; *N* is neural clique numbers, whereas *i* is distinct information; Figure 1C; Tsien, 2015a,b; Li et al., 2016). The proposed power-of-two-based computational logic has been observed in at least seven different brain regions, ranging from the amygdala to the hippocampus to the cortex in mice and hamsters in the form of cell-assembly activation patterns (Xie et al., 2016; Figure 1D). In contrast to Hebb's postulate that cell assembly is formed by learning, this basic logic is pre-configured by development since the logic remains largely intact in the NMDA receptor knockout mice (Xie et al., 2016). Due to the power-of-two-based logic, as the number of distinct-information inputs increase, the number of principal cells required to construct FCMs can grow dramatically. For example, at least 1,023 principal-projection cells would be required if the functional connectivity motif were set to process 10 distinct features or events (assuming one neuron per clique, according to the *N* = 2^*i*^ -1 equation). As categorically distinct inputs increase to 15, the minimal cell numbers would be 32,383.

If one neuron were to have only several dozen synapses merely for information integration and binding, such a narrow bandwidth would mean that this entire FCM would unlikely be used in a lifetime of animals unless information from peripheral sensors could match exactly with these cells' tuning properties. On the other hand, the utility of the power-of-two-based FCM logic can be best realized via dramatically increasing the number of synapses per neuron so that the approximation capacity—or degree in detecting similar features or categorical variables—can be dramatically expanded (Tsien, 2016). At the structural level, synapses processing similar variables can be localized on the same dendritic branches (Lai et al., 2012) or different branches (Basu et al., 2016). In any case, with tens of thousands of synapses per neuron, the FCM can be best structured not only for information-binding (which can be largely taken care of by the FCM's specific-to-general neural cliques), but also for maximal approximation and utilization. It would be of considerable interest to examine how synapse numbers and spike variability may correlate.

## How do rate code, temporal code and population code deal with neuronal variability?

The *rate code* refers to the notion that information about the stimulus is encoded by the firing rate of the neuron (Figure 1E). In practice, the rate is measured by averaging the number of spikes per second or a defined (often smaller) time bin before and after stimulus presentation and typically over multiple stimulus trials. This averaging procedure inherently assumes that spike variability reflects noise—and most, if not all, information is conveyed by spike numbers. Any information possibly encoded in the temporal structure of the spike train is purposely ignored (Figure 1E). While the rate code model is convenient for researchers to define the tuning properties of the neurons by averaging spike responses over multiple trials, the brain is unlikely to generate perception, memory or action in real time using this procedure.

To potentially overcome such neuronal variability of individual neurons, researchers have applied a population vector or dimensionality-reduction classification methods to analyze the rate code information using the population activity of many neurons (Georgopoulos et al., 1986; Wilson and Mcnaughton, 1993; Lin et al., 2005; Chen et al., 2009; Luczak et al., 2015). Conceptually, neurons with similar tuning properties, termed neural cliques, can be temporally averaged to reduce individual variability (Lin et al., 2005, 2006; Zhang et al., 2013; Luczak et al., 2015). It should be noted that the population-code approach has allowed researchers to sidestep the problem of neuronal variability as observed at the single neuron level, yet the underlying assumption that neuronal variability is noise still remains the same.

The second major type of the proposed neural coding model is referred to as the *temporal code*, which utilizes timing information of spike discharges to signal the stimulus identity [for a classic review, see (Perkel and Bullock, 1968; Theunissen and Miller, 1995; Singer, 1999)]. There are at least three models in the literature that offer different ideas about how to use time information (Figures 1F–H). The first classic temporal-code model is the “*time-to-first-spike*” model (Figure 1F). In sensory neurons, several laboratories reported that most of the information about a stimulus is conveyed during the first 20~50 ms after the onset of the cell response (Optican and Richmond, 1987; Tovee et al., 1993; Kjaer et al., 1994; Tovee and Rolls, 1995). This *time-to-first-spiking* model requires the experimental observers to set the time reference points (i.e., time zeros of stimulation) for data analysis. Because spike discharge is a continuous process, a neuron sitting inside the brain would not have the leverage to know which spike is the first spike and who should be doing the counting of these *special* spikes.

The second temporal coding model is the “*spike*-*phase code*” which uses a periodic signal, such as the specific phase of local field potential (LFP) oscillations, as the reference signal (Figure 1G). For example, decoding reliability of odor identity or an animal's spatial location can be increased if one combines changes in spike rates with a selectively filtered LFP oscillation phase (O'keefe and Recce, 1993; Hopfield, 1995; Jensen and Lisman, 1996; Wehr and Laurent, 1996). In practice, the spike phase-coupling code often involved the data averaging of stimulus-triggered spikes over the trials, like the rate-code model, and then aligning them with phases of LFP oscillation. Thus, this temporal coding model still treats spike variability as noise that degrades decoding accuracy. Behaviorally, oscillations seem to reflect ongoing motor activity such as respiration (used for analyzing odor cell-phase coupling) or running (used for analyzing CA1 place cell-phase coupling). In addition, membrane oscillations are well known to be highly variable over the spatial domain (i.e., dendrites vs. soma), as well as the temporal domain. To reduce variability that corrupts tuning properties of cells, several artificial manipulations were often employed. For instance, place-cell analyses were usually limited to the locomotion state during which animals need to reach a certain running speed; any spike data occurring during a momentary pause, grooming, eating, or below the defined running speed were artificially excluded, artificially excluded, a procedure which the brain is unlikely to do. Another unresolved issue is that LFPs are intermixed voltage signals that do not separate themselves neatly into different frequency bands. As such, it is unclear how neurons would filter out specific oscillations (i.e., theta or gamma frequencies) and then perform spike phase coupling analysis as experimenters did via Matlab software.

The third type of temporal coding models is the *spike-synchrony code* (Figure 1H). It refers to the hypothesis that neurons encode information about the stimulus identity by modulating not only the firing rate of individual neurons, but also by temporally synchronized spiking across different neurons. Studies from the primary auditory cortex, retina and primary visual cortex found precise firing responses to auditory or visual stimuli, respectively, with millisecond precision from trial to trial [see a comprehensive review, (Singer, 1999)]. Highly synchronous oscillatory discharges of retinal responses that reach oscillation frequencies of up to 100 Hz are also transmitted reliably from the retina to the primary visual cortex (Neuenschwander and Singer, 1996; Castelo-Branco et al., 1998; Herculano-Houzel et al., 1999; Neuenschwander et al., 1999). In addition, spike synchrony has been proposed for binding or grouping to achieve dynamic and flexibility of coding. Still, analysis of these binding operations requires measurements of temporal relations among distributed cell responses that will need more sophisticated statistical procedures than pairwise correlations. While correlation analysis can be useful in measuring degrees of spike synchrony, fluctuating spike-discharge patterns can easily overwhelm and corrupt synchrony-based code (Softky and Koch, 1993; Stevens and Zador, 1998; Shadlen and Movshon, 1999).

## Self-information code: variability is the self-information expressor

**Figure 2:**
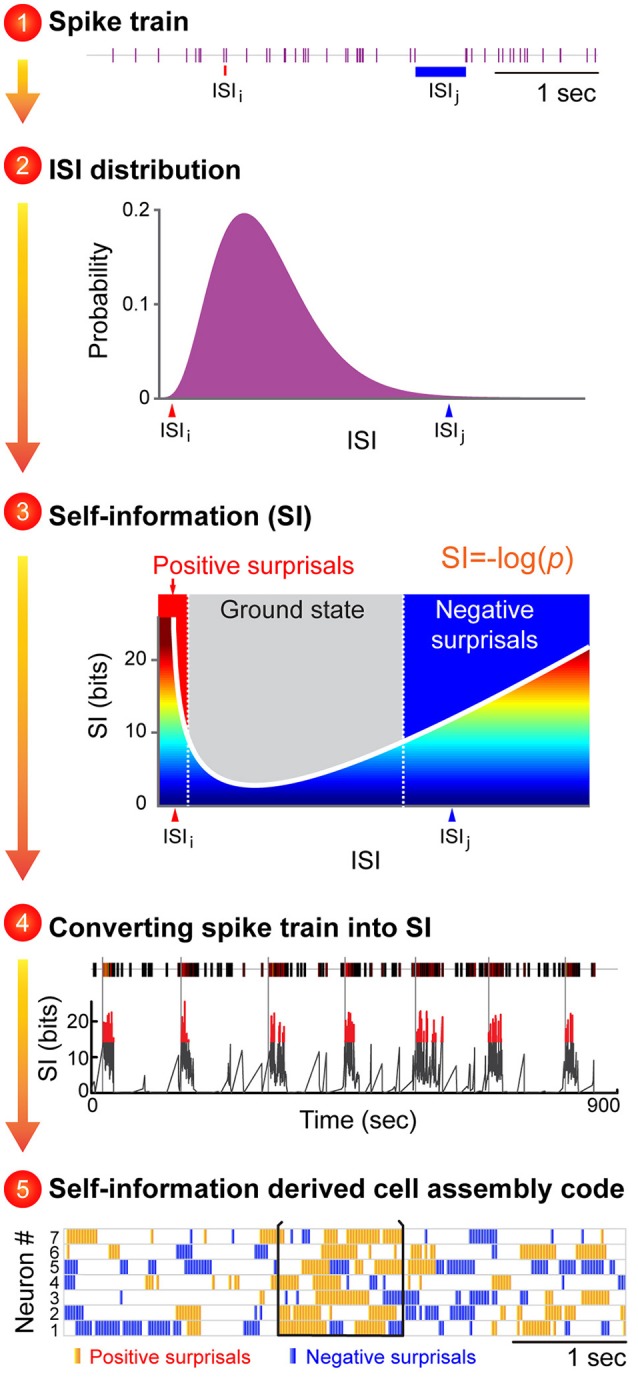
An illustration to describe how the proposed Neural Self-Information Theory can be used to decode cell-assembly patterns from neuronal spike trains. The *Self-Information Code* is proposed to explain how real-time neural code is generated via spike-timing variability, and how cell-assembly patterns can be identified in an unbiased manner. A general strategy to apply the neural self-information theory to uncover cell assemblies from spike-train datasets. Cell-assembly code is identified in four steps based on the conversion of an individual neuron's spike train into variability distribution of ISIs, followed by its conversion to a real-time self-information value. The temporally coordinated self-information surprisal patterns across cell population can be detected in an unbiased manner by pattern classification methods such as blind-source analyses. The unique feature of this self-information code is that this neural coding principle is completely intrinsic to the neurons themselves, with no need for any reference point to be set by outside observers.

Here we present the *Neural Self-Information Theory* that neural coding is a self-information process based on inter-spike-interval (neuronal silence duration) variability and its variability history. Contrary to the prevalent view that spike variability reflects noise or is merely correlated with some unknown modulatory variables, we postulate that neuronal variability is what carries information by itself. Specifically, information is encoded by utilizing real-time inter-spike-interval (ISI) variability under the probability-based statistical self-information principles. According to this neural self-information theory, neuronal variability operates as the self-information generator and expressor; higher-probability ISIs, which reflect the balanced excitation-inhibition ground-state, convey less information, whereas lower-probability ISIs (the rare-occurrence of silence durations, such as unusually brief or prolonged ISIs), which signify statistical surprisals, convey more information (Figure 2A).

In other words, under this *Self-Information Code*, any given ISI is self-tagged with a discrete amount of information based on its silence duration in a relationship with the silence-duration probability-distribution history (Figure 2). This probability distribution of ISI fluctuations or neuronal silence time-duration is intrinsically sensed by and coupled with ongoing biochemical reactions and energy equilibrium. For example, a prolonged neuronal silence requires less ATP consumption, whereas the production of multiple extremely short ISIs which reflect strong spike bursting requires more ATP. Thus, this silence-based self-information process is naturally unified with intracellular biochemical reaction cascades, including receptor turnover, protein endocytosis, and new protein synthesis and gene expression.

At the physiological level, these self-information ISI surprisals can be either positive surprisals (when a neuron's ISI becomes much shorter than is typical, reflecting strong excitation) or negative surprisals (when ISI becomes much longer than is typical, reflecting strong inhibition; Figure 2, see Step 3). In practice, the measurement of ISI probability distribution can be determined easily. In contrast to the prevalent notion that spike variability is a Poisson distribution, emerging studies have suggested that ISI variability in many neural circuits conform to the gamma distribution (Li et al., 2017). As such, instead of measuring the mean value of the information contained in a spike train (namely, the information entropy), each ISI can be precisely calculated for its information content (*I*) based on a simple self-information equation [*I* = −log(*P*), where *P* is the probability of each ISI (the time period between two successful spikes)]. In statistical term, those events with low-occurrence probability is called *surprisals*. Subsequently, these dynamic, transient surprisal ISI patterns would act as the critical real-time information packets at a single neuron level. When these surprisal ISIs are emitted across a cell population in a temporally coordinated manner, they can seamlessly give rise to robust real-time cell-assembly code.

The variability-surprisal-based neural self-information theory makes several testable predictions: if neuronal variability acts as a self-information generator, this variability should remain similar across various brain regions. On the other hand, if neuronal variability reflects system noise, one would expect that variability would grow larger as information is transmitted from low subcortical structures to the high-cognition cortices.

To differentiate these two scenarios, one can record large numbers of neurons from various cortical and sub-cortical regions in freely behaving animals. To facilitate systematic comparisons, one can initially focus on putatively classified principal cells after these units have been separated from fast-spiking putative interneurons and their variability distributions analyzed across these different regions. In addition, to examine the potential state-dependent influence on neuronal variability, one can assess the spike datasets collected from the quiet-awake state as animals rested in their home-cage environments, and compare them with those patterns obtained during various cognitive tasks.

One can characterize neuronal variability by using three well-defined statistics to describe quantitatively neuronal variability of a neuron's ISI—namely, a coefficient of variation (CV), skewness and kurtosis. In probability theory and statistics, CV is a standardized measure of dispersion of a probability distribution, and skewness is a measure of the asymmetry of a probability distribution, whereas kurtosis is a measure of the “tailedness” of a probability distribution. We would predict that principal cells in various brain regions should exhibit similar neuronal-variability distributions. To further test the idea that neuronal variability serves as a self-information carrier, we also predicted that variability would diminish under the condition that both external and internal neural computations were artificially shut down (i.e., upon anesthesia). Pharmacological intervention experiments can be used to demonstrate that the shutting down of external and internal coding processes would indeed greatly reduce neuronal variability. If so, it would be consistent with the notion that spike variability reflects the ongoing cognitive processing of both external and internal information.

## A general decoding strategy to uncover various cell assemblies

One immediate application of this “self-information code” concept is that it should enable researchers to identify a variety of cell assemblies from large-scale recording datasets. Overall, this variability-surprisal-based, cell-assembly decoding (VCAD) strategy can consist of the following four major steps (Figure 2):

The first step is to convert each neuron's spike train into the probability distribution of ISI variability. Since ISI patterns in various neural circuits seem to conform to the gamma distribution model (Li et al., 2017), one can be achieved by fitting a single neuron's ISI with a gamma distribution model which can assign each neuron's ISI with a probability.

The second step is to convert the probability distribution of ISI variability into real-time self-information distribution for each ISI. These frequent ISI variations with high probability represent the low self-information or ground state (Figure 2, subtitle #3, the gray zone in the mid-section of the Self-information plot). As a neuron increased its firing, it generates positive surprisals (the red curve inside the Self-information plot) as ISIs entered the left tail zone of the distribution probability (a low-probability state). On the other hand, if the neuron's firing is dramatically suppressed, negative surprisals are generated (the blue curve inside the Self-information plot) when ISIs shifted to the right tail zone (also a low-probability state).

In the third step, a spike train emitted by a neuron is transformed into a surprisal-based self-information code using the dynamic evolution of ISI patterns (silence time patterns). The time-window for practical estimation of the instantaneous ISI distribution for each neuron may depend on the averaged firing rate, with low firing neuron requiring longer time duration (i.e., 15 min) and high-firing neuron requiring shorter time duration. Biologically speaking, the time durations for defining the probability distribution patterns may be best examined by measuring the molecular and synaptic turnover rates or other key biochemical processes which reflect the intracellular memory time-scale of neuronal equilibrium.

The fourth step is to uncover joint surprisal-spike patterns across simultaneously-recorded cells on a moment-to-moment basis. Blind-source-separation (BSS) methods, such as independent component analysis (ICA), can identify a set of independent information sources from simultaneously observed signals such as structured patterns or relationships. Each independent signal source decoded by BSS would correspond to a distinct real-time activation pattern given by a cell assembly.

To discover its functional meaning, one can compare each real-time activation temporal pattern with various other experimental parameters [such as the dynamic evolution of local field potential (LFP), the time points of stimulus presentations, videotapes of an animal's behavioral state, actions, and corresponding locations, etc.].

Moreover, the top-ranking membership with the highest contribution weights in the cell assembly can be directly identified from demixing matrix W. This will allow researchers to assess quantitative membership information that other dimensionality-reduction-based, pattern-classification methods (i.e., principal component analysis or multiple discriminant analysis) could not provide. By further mapping cell-assembly activity patterns onto specific cell types and network states (Klausberger and Somogyi, 2008; Colgin, 2016; Van De Ven et al., 2016), we expect that researchers can gain greater insights into how neural code is generated within and across the evolutionarily conserved computational motifs (Grillner, 2006; Brenner and Sejnowski, 2011; Marcus et al., 2014; Tsien, 2015a,b; Kiehn, 2016; Xie et al., 2016).

In summary, we present a new hypothesis on how to crack the real-time neural code. Specifically, we put forth the *Neural Self-Information Theory* that neuronal variability operates as the self-information generator and expressor to convey a variable amount of information in the form of silence variability-surprisals. Coordination of these surprisal ISIs in space (across cells) and time can seamlessly give rise to robust real-time cell-assembly code. It should be noted that while the ground state corresponds to the most probable ISI which carry less self-information (and yet may consume a lot of energy during non-coding state), they can be extremely important in terms of providing both the rapid responses to changes and ternary coding structure once combined with positive and negative surprisals, which leads to enormous information capacity and flexibility at the cell-assembly level. The generality of this *silence-based self-information code* can be demonstrated by identifying real-time cell assemblies processing internal states, external experiences—including continuous variables—and categorical variables. Most importantly, this *Self-Information Code* is completely intrinsic to neurons themselves, with no need for outside observers to set any reference point such as time zeros of stimulation or filtered local field potential oscillation phases. Because the self-information code is operated in the form of the ISI variability-based probability distribution, the downstream neurons can naturally sense these surprisal shift in ISI variability as manifested by sudden deviations from the equilibrium (a form of intracellular memory or variability distribution) of post-synaptic neurons' biochemical states (i.e., energy production, receptor activation, ion channel open/close state distribution patterns, protein phosphorylation/dephosphorylation rate, receptor insertion/removal, etc.).

In light of the theoretical and practical implications, the proposed *Neural Self-Information* Theory can be examined via large-scale *in vivo* recording experiments. Moreover, this silence-duration varibility-based surprisal coding concept can also be exploited for the design of a novel neuromorphic chip for brain-inspired computing with robust resilience to interference. As Wolf Singer once noted, “We nonetheless shall have made a great step forward, because *it is the unexpected result that contains maximal information*.”

## Ethics statement

All animal work described in the study was carried out in accordance with the guidelines laid down by the National Institutes of Health in the United States, regarding the care and use of animals for experimental procedures, and was approved by the Institutional Animal Care and Use Committee of Augusta University (Approval AUP number: BR07-11-001).

## Author contributions

ML and JT conceived the idea. JT wrote the paper with ML.

### Conflict of interest statement

The authors declare that the research was conducted in the absence of any commercial or financial relationships that could be construed as a potential conflict of interest. The handling Editor currently co-hosts a Research Topic with one of the authors JT, and confirms the absence of any other collaboration.

## References

[B1] Abbott L. Sejnowski T. J. (1999). Neural Codes and Distributed Representations: Foundations of Neural Computation. Cambridge, MA: MIT Press.

[B2] Adrian E. D. Zotterman Y. (1926). The impulses produced by sensory nerve endings: part 3. Impulses set up by Touch and Pressure. J. Physiol. 61, 465–483. 10.1113/jphysiol.1926.sp002308 16993807 PMC1514868

[B3] Andersen P. (1990). Synaptic integration in hippocampal CA1 pyramids. Prog. Brain Res. 83, 215–222. 10.1016/S0079-6123(08)61251-0 2168057

[B4] Basu J. Zaremba J. D. Cheung S. K. Hitti F. L. Zemelman B. V. Losonczy A.. (2016). Gating of hippocampal activity, plasticity, and memory by entorhinal cortex long-range inhibition. Science 351:aaa5694. 10.1126/science.aaa5694 26744409 PMC4920085

[B5] Boerlin M. Deneve S. (2011). Spike-based population coding and working memory. PLoS Comput. Biol. 7:e1001080. 10.1371/journal.pcbi.1001080 21379319 PMC3040643

[B6] Brenner R. Chen Q. H. Vilaythong A. Toney G. M. Noebels J. L. Aldrich R. W. (2005). BK channel beta4 subunit reduces dentate gyrus excitability and protects against temporal lobe seizures. Nat. Neurosci. 8, 1752–1759. 10.1038/nn1573 16261134

[B7] Brenner S. Sejnowski T. J. (2011). Understanding the human brain. Science 334:567 10.1126/science.1215674 PMC4757457 22053011

[B8] Brown E. N. Kass R. E. Mitra P. P. (2004). Multiple neural spike train data analysis: state-of-the-art and future challenges. Nat. Neurosci. 7, 456–461. 10.1038/nn1228 15114358

[B9] Castelo-Branco M. Neuenschwander S. Singer W. (1998). Synchronization of visual responses between the cortex, lateral geniculate nucleus, and retina in the anesthetized cat. J. Neurosci. 18, 6395–6410. 9698331 10.1523/JNEUROSCI.18-16-06395.1998 PMC6793201

[B10] Chang L. Tsao D. Y. (2017). The code for facial identity in the primate brain. Cell 169, 1013–1028.e1014. 10.1016/j.cell.2017.05.011 28575666 PMC8088389

[B11] Chen G. Wang L. P. Tsien J. Z. (2009). Neural population-level memory traces in the mouse hippocampus. PLoS ONE 4:e8256. 10.1371/journal.pone.0008256 20016843 PMC2788416

[B12] Churchland M. M. Yu B. M. Cunningham J. P. Sugrue L. P. Cohen M. R. Corrado G. S. (2010). Stimulus onset quenches neural variability: a widespread cortical phenomenon. Nat. Neurosci. 13, 369–378. 10.1038/nn.2501 20173745 PMC2828350

[B13] Colgin L. L. (2016). Rhythms of the hippocampal network. Nat. Rev. Neurosci. 17, 239–249. 10.1038/nrn.2016.21 26961163 PMC4890574

[B14] Defelipe J. (2015). The anatomical problem posed by brain complexity and size: a potential solution. Front. Neuroanat. 9:104. 10.3389/fnana.2015.00104 26347617 PMC4542575

[B15] Eggermont J. J. (1998). Is there a neural code? Neurosci. Biobehav. Rev. 22, 355–370. 10.1016/S0149-7634(97)00021-3 9579325

[B16] Ermentrout G. B. Galan R. F. Urban N. N. (2008). Reliability, synchrony and noise. Trends Neurosci. 31, 428–434. 10.1016/j.tins.2008.06.002 18603311 PMC2574942

[B17] Faisal A. A. Selen L. P. Wolpert D. M. (2008). Noise in the nervous system. Nat. Rev. Neurosci. 9, 292–303. 10.1038/nrn2258 18319728 PMC2631351

[B18] Fenton A. A. Muller R. U. (1998). Place cell discharge is extremely variable during individual passes of the rat through the firing field. Proc. Natl. Acad. Sci. U.S.A. 95, 3182–3187. 10.1073/pnas.95.6.3182 9501237 PMC19716

[B19] Georgopoulos A. P. Schwartz A. B. Kettner R. E. (1986). Neuronal population coding of movement direction. Science 233, 1416–1419. 10.1126/science.3749885 3749885

[B20] Grillner S. (2006). Biological pattern generation: the cellular and computational logic of networks in motion. Neuron 52, 751–766. 10.1016/j.neuron.2006.11.008 17145498

[B21] Grillner S. Robertson B. Stephenson-Jones M. (2013). The evolutionary origin of the vertebrate basal ganglia and its role in action selection. J. Physiol. 591, 5425–5431. 10.1113/jphysiol.2012.246660 23318875 PMC3853485

[B22] Gross C. G. Lettvin J. Konorski J. (2002). Genealogy of the “grandmother cell.” Neuroscientist 8, 512–518. 10.1177/107385802237175 12374433

[B23] Gu N. Vervaeke K. Storm J. F. (2007). BK potassium channels facilitate high-frequency firing and cause early spike frequency adaptation in rat CA1 hippocampal pyramidal cells. J. Physiol. 580, 859–882. 10.1113/jphysiol.2006.126367 17303637 PMC2075463

[B24] Guillery R. W. (2005). Observations of synaptic structures: origins of the neuron doctrine and its current status. Philos. Trans. R. Soc. Lond. Ser. B. Biol. Sci. 360, 1281–1307. 10.1098/rstb.2003.1459 16147523 PMC1569502

[B25] Hartmann C. Lazar A. Nessler B. Triesch J. (2015). Where's the noise? Key features of spontaneous activity and neural variability arise through learning in a deterministic network. PLoS Comput. Biol. 11:e1004640. 10.1371/journal.pcbi.1004640 26714277 PMC4694925

[B26] Hebb D. O. (1949). The Organization of Behavior: A Neuropsychological Approach. New York, NY: John Wiley & Sons.

[B27] Herculano-Houzel S. (2009). The human brain in numbers: a linearly scaled-up primate brain. Front. Hum. Neurosci. 3:31. 10.3389/neuro.09.031.2009 19915731 PMC2776484

[B28] Herculano-Houzel S. Munk M. H. Neuenschwander S. Singer W. (1999). Precisely synchronized oscillatory firing patterns require electroencephalographic activation. J. Neurosci. 19, 3992–4010. 10234029 10.1523/JNEUROSCI.19-10-03992.1999 PMC6782718

[B29] Hille B. (2001). Ion Channels of Excitable Membranes. Sunderland: Sinauer.

[B30] Hopfield J. J. (1995). Pattern recognition computation using action potential timing for stimulus representation. Nature 376, 33–36. 10.1038/376033a0 7596429

[B31] Jensen O. Lisman J. E. (1996). Hippocampal CA3 region predicts memory sequences: accounting for the phase precession of place cells. Learn. Mem. 3, 279–287. 10.1101/lm.3.2-3.279 10456097

[B32] Kiehn O. (2016). Decoding the organization of spinal circuits that control locomotion. Nat. Rev. Neurosci. 17, 224–238. 10.1038/nrn.2016.9 26935168 PMC4844028

[B33] Kjaer T. W. Hertz J. A. Richmond B. J. (1994). Decoding cortical neuronal signals: network models, information estimation and spatial tuning. J. Comput. Neurosci. 1, 109–139. 10.1007/BF00962721 8792228

[B34] Klausberger T. Somogyi P. (2008). Neuronal diversity and temporal dynamics: the unity of hippocampal circuit operations. Science 321, 53–57. 10.1126/science.1149381 18599766 PMC4487503

[B35] Kohn A. Coen-Cagli R. Kanitscheider I. Pouget A. (2016). Correlations and Neuronal Population Information. Ann. Rev. Neurosci. 39, 237–256. 10.1146/annurev-neuro-070815-013851 27145916 PMC5137197

[B36] Lai C. S. W. Franke T. F. Gan W.-B. (2012). Opposite effects of fear conditioning and extinction on dendritic spine remodelling. Nature 483, 87–91. 10.1038/nature10792 22343895

[B37] Lee D. Port N. L. Kruse W. Georgopoulos A. P. (1998). Variability and correlated noise in the discharge of neurons in motor and parietal areas of the primate cortex. J. Neurosci. 18, 1161–1170. 9437036 10.1523/JNEUROSCI.18-03-01161.1998 PMC6792758

[B38] Li M. Liu J. Tsien J. Z. (2016). Theory of connectivity: nature and nurture of cell assemblies and cognitive computation. Front. Neural Circuits 10:34 10.3389/fncir.2016.00034 PMC4850152 27199674

[B39] Li M. Xie K. Kuang H. Liu J. Wang D. Fox G. E. (2017). Spike-timing patterns conform to gamma distribution with regional and cell type-specific characteristics. Biorxiv 145813 10.1101/145813

[B40] Lin I. C. Okun M. Carandini M. Harris K. D. (2015). The nature of shared cortical variability. Neuron 87, 644–656. 10.1016/j.neuron.2015.06.035 26212710 PMC4534383

[B41] Lin L. Chen G. Kuang H. Wang D. Tsien J. Z. (2007). Neural encoding of the concept of nest in the mouse brain. Proc. Natl. Acad. Sci. U.S.A. 104, 6066–6071. 10.1073/pnas.0701106104 17389405 PMC1851617

[B42] Lin L. Chen G. Xie K. Zaia K. A. Zhang S. Tsien J. Z. (2006). Large-scale neural ensemble recording in the brains of freely behaving mice. J. Neurosci. Methods 155, 28–38. 10.1016/j.jneumeth.2005.12.032 16554093

[B43] Lin L. Osan R. Shoham S. Jin W. Zuo W. Tsien J. Z. (2005). Identification of network-level coding units for real-time representation of episodic experiences in the hippocampus. Proc. Natl. Acad. Sci. U.S.A. 102, 6125–6130. 10.1073/pnas.0408233102 15833817 PMC1087910

[B44] Luczak A. Mcnaughton B. L. Harris K. D. (2015). Packet-based communication in the cortex. Nat. Rev. Neurosci. 16, 745–755. 10.1038/nrn4026 26507295

[B45] Mainen Z. F. Sejnowski T. J. (1995). Reliability of spike timing in neocortical neurons. Science 268, 1503–1506. 10.1126/science.7770778 7770778

[B46] Marcos E. Pani P. Brunamonti E. Deco G. Ferraina S. Verschure P. (2013). Neural variability in premotor cortex is modulated by trial history and predicts behavioral performance. Neuron 78, 249–255. 10.1016/j.neuron.2013.02.006 23622062

[B47] Marcus G. Marblestone A. Dean T. (2014). The atoms of neural computation. Science 346, 551–552. 10.1126/science.1261661 25359953

[B48] Masquelier T. (2013). Neural variability, or lack thereof. Front. Comput. Neurosci. 7:7 10.3389/fncom.2013.00007 23444270 PMC3580760

[B49] Megias M. Emri Z. Freund T. F. Gulyas A. I. (2001). Total number and distribution of inhibitory and excitatory synapses on hippocampal CA1 pyramidal cells. Neuroscience 102, 527–540. 10.1016/S0306-4522(00)00496-6 11226691

[B50] Neuenschwander S. Singer W. (1996). Long-range synchronization of oscillatory light responses in the cat retina and lateral geniculate nucleus. Nature 379, 728–732. 10.1038/379728a0 8602219

[B51] Neuenschwander S. Castelo-Branco M. Singer W. (1999). Synchronous oscillations in the cat retina. Vision Res. 39, 2485–2497. 10.1016/S0042-6989(99)00042-5 10396618

[B52] O'keefe J. Recce M. L. (1993). Phase relationship between hippocampal place units and the EEG theta rhythm. Hippocampus 3, 317–330. 10.1002/hipo.450030307 8353611

[B53] Optican L. M. Richmond B. J. (1987). Temporal encoding of two-dimensional patterns by single units in primate inferior temporal cortex. III. Information theoretic analysis. J. Neurophysiol. 57, 162–178. 3559670 10.1152/jn.1987.57.1.162

[B54] Perkel D. H. Bullock T. H. (1968). Neural coding. Neurosci. Res. Prog. Bull. 3, 405–527.

[B55] Poggio G. F. Viernstein L. J. (1964). Time series analysis of impulse sequences of thalamic somatic sensory neurons. J. Neurophysiol. 27, 517–545. 14194957 10.1152/jn.1964.27.4.517

[B56] Quiroga R. Q. Reddy L. Kreiman G. Koch C. Fried I. (2005). Invariant visual representation by single neurons in the human brain. Nature 435, 1102–1107. 10.1038/nature03687 15973409

[B57] Rakic P. (2009). Evolution of the neocortex: a perspective from developmental biology. Nat. Rev. Neurosci. 10, 724–735. 10.1038/nrn2719 19763105 PMC2913577

[B58] Ratliff F. Hartline H. K. Lange D. (1968). Variability of interspike intervals in optic nerve fibers of Limulus: effect of light and dark adaptation. Proc. Natl. Acad. Sci. 60, 464–469. 10.1073/pnas.60.2.464 5248805 PMC225070

[B59] Rolls E. Deco G. (2010). The Noisy Brain. Stochastic Dynamics As a Principle of Brain Function. London: Oxford University Press. 10.1016/j.pneurobio.2009.01.006 19428958

[B60] Saberi-Moghadam S. Ferrari-Toniolo S. Ferraina S. Caminiti R. Battaglia-Mayer A. (2016). Modulation of neural variability in premotor, motor, and posterior parietal cortex during change of motor intention. J. Neurosci. 36, 4614–4623. 10.1523/JNEUROSCI.3300-15.2016 27098702 PMC6601830

[B61] Shadlen M. N. Movshon J. A. (1999). Synchrony unbound: a critical evaluation of the temporal binding hypothesis. Neuron 24, 67–77, 111–125. 10.1016/S0896-6273(00)80822-3 10677027

[B62] Shadlen M. N. Newsome W. T. (1994). Noise, neural codes and cortical organization. Curr. Opin. Neurobiol. 4, 569–579. 10.1016/0959-4388(94)90059-0 7812147

[B63] Singer W. (1999). Neuronal synchrony: a versatile code for the definition of relations? Neuron 24, 49–65, 111–125. 10.1016/S0896-6273(00)80821-1 10677026

[B64] Softky W. R. Koch C. (1993). The highly irregular firing of cortical cells is inconsistent with temporal integration of random EPSPs. J. Neurosci. 13, 334–350. 8423479 10.1523/JNEUROSCI.13-01-00334.1993 PMC6576320

[B65] Squire L. R. Zola S. M. (1998). Episodic memory, semantic memory, and amnesia. Hippocampus 8, 205–211. 9662135 10.1002/(SICI)1098-1063(1998)8:3<205::AID-HIPO3>3.0.CO;2-I

[B66] Stacey W. C. Durand D. M. (2001). Synaptic noise improves detection of subthreshold signals in hippocampal CA1 neurons. J. Neurophysiol. 86, 1104–1112. 11535661 10.1152/jn.2001.86.3.1104

[B67] Stein R. B. Gossen E. R. Jones K. E. (2005). Neuronal variability: noise or part of the signal? Nat. Rev. Neurosci. 6, 389–397. 10.1038/nrn1668 15861181

[B68] Stevens C. F. Zador A. M. (1998). Input synchrony and the irregular firing of cortical neurons. Nat. Neurosci. 1, 210–217. 10.1038/659 10195145

[B69] Theunissen F. Miller J. P. (1995). Temporal encoding in nervous systems: a rigorous definition. J. Comput. Neurosci. 2, 149–162. 10.1007/BF00961885 8521284

[B70] Toups J. V. Fellous J.-M. Thomas P. J. Sejnowski T. J. Tiesinga P. H. (2012). Multiple spike time patterns occur at bifurcation points of membrane potential dynamics. PLoS Comput. Biol. 8:e1002615. 10.1371/journal.pcbi.1002615 23093916 PMC3475656

[B71] Tovee M. J. Rolls E. T. (1995). Information encoding in short firing rate epochs by single neurons in the primate temporal visual cortex. Visual Cogn. 2, 35–58. 10.1080/13506289508401721

[B72] Tovee M. J. Rolls E. T. Treves A. Bellis R. P. (1993). Information encoding and the responses of single neurons in the primate temporal visual cortex. J. Neurophysiol. 70, 640–654. 8410164 10.1152/jn.1993.70.2.640

[B73] Tsien J. Z. (2007). The memory code. Sci. Am. 297, 52–59. 10.1038/scientificamerican0707-52 17695842

[B74] Tsien J. Z. (2015a). A postulate on the brain's basic wiring logic. Trends Neurosci. 38, 669–671. 10.1016/j.tins.2015.09.002 26482260 PMC4920130

[B75] Tsien J. Z. (2015b). Principles of intelligence: on evolutionary logic of the brain. Front. Syst. Neurosci. 9:186. 10.3389/fnsys.2015.00186 26869892 PMC4739135

[B76] Tsien J. Z. (2016). Cre-Lox Neurogenetics: 20 years of versatile applications in brain research and counting. Front. Genet. 7:19. 10.3389/fgene.2016.00019 26925095 PMC4759636

[B77] Tsien J. Z. Li M. Osan R. Chen G. Lin L. Wang P. L.. (2013). On initial brain activity mapping of episodic and semantic memory code in the hippocampus. Neurobiol. Learn. Memory 105, 200–210. 10.1016/j.nlm.2013.06.019 23838072 PMC3769419

[B78] Tulving E. (1972). Episodic and semantic memory, in Organization of Memory, eds Tulving E. Donaldson W. (New York, NY: Academic Press), 381–403.

[B79] Van De Ven G. M. Trouche S. Mcnamara C. G. Allen K. Dupret D. (2016). Hippocampal offline reactivation consolidates recently formed cell assembly patterns during sharp wave-ripples. Neuron. 92, 968–974. 10.1016/j.neuron.2016.10.020 27840002 PMC5158132

[B80] Wehr M. Laurent G. (1996). Odour encoding by temporal sequences of firing in oscillating neural assemblies. Nature 384, 162–166. 10.1038/384162a0 8906790

[B81] Werner G. Mountcastle V. B. (1963). The variability of central neural activity in a sensory system, and its implications for the central reflection of sensory events. J. Neurophysiol. 26, 958–977. 14084169 10.1152/jn.1963.26.6.958

[B82] Wilson M. A. Mcnaughton B. L. (1993). Dynamics of the hippocampal ensemble code for space. Science 261, 1055–1058. 10.1126/science.8351520 8351520

[B83] Xie K. Fox G. E. Liu J. Lyu C. Lee J. C. Kuang H.. (2016). Brain Computation is organized via power-of-two-based permutation logic. Front. Syst. Neurosci. 10:95. 10.3389/fnsys.2016.00095 27895562 PMC5108790

[B84] Zhang H. Chen G. Kuang H. Tsien J. Z. (2013). Mapping and deciphering neural codes of NMDA receptor-dependent fear memory engrams in the hippocampus. PLoS ONE 8:e79454. 10.1371/journal.pone.0079454 24302990 PMC3841182

